# Impact of Commercial Oenotannin and Mannoprotein Products on the Chemical and Sensory Properties of Shiraz Wines Made from Sequentially Harvested Fruit

**DOI:** 10.3390/foods7120204

**Published:** 2018-12-12

**Authors:** Sijing Li, Keren Bindon, Susan Bastian, Kerry Wilkinson

**Affiliations:** 1 School of Agriculture, Food and Wine, The University of Adelaide, Waite Campus, PMB 1, Glen Osmond, SA 5064, Australia; sli@csu.edu.au (S.L.); sue.bastian@adelaide.edu.au (S.B.); 2 The Australian Research Council Training Centre for Innovative Wine Production, PMB 1, Glen Osmond, SA 5064, Australia; 3 The Australian Wine Research Institute, P.O. Box 197, Glen Osmond, SA 5064, Australia; Keren.Bindon@awri.com.au

**Keywords:** additives, mannoprotein, polysaccharides, sensory evaluation, tannin, wine

## Abstract

The tannin and polysaccharide profiles and therefore sensory properties of wine are influenced by fruit maturity at harvest, and practices employed during winemaking. This study investigated the extent to which commercial winemaking supplements (skin and seed tannins, and mannoprotein (MP)) can enhance the mouthfeel properties of red wine, in particular, wine made from grapes harvested before commercial ripeness (early-harvest). Supplements were added to wines made from Shiraz grapes harvested at 20.8 and 24.5 °Brix. The chemical composition and mouthfeel properties of wines were then determined by high performance liquid chromatography and descriptive analysis (DA), respectively. Wines made from riper grapes had higher levels of tannin than wines made from early-harvest grapes, but similar polysaccharide levels were observed. The addition of seed oenotannin yielded higher tannin levels than addition of skin oenotannin, particularly for wines made from early-harvest grapes. The DA panel perceived sensory differences between H1 and H2 wines, but could not perceive any effect of supplementation on wine mouthfeel properties, with the exception of a minor increase in sweetness, attributed to mannoprotein addition to H1 wines, even when MP was added to wines at 2.5 times the level recommended for use in Australia.

## 1. Introduction

The alcoholic strength of table wines has increased considerably since the 1990s in all major wine producing countries [1]. This trend is considered an inadvertent effect of increased sugar levels in grapes at harvest (which is attributable to many factors, including warmer climates and healthier vines), yeast strains which more efficiently convert sugar to ethanol, and the popularity of full-bodied, fruit-driven wine styles [2]. However, increased grape sugar levels can pose significant challenges for fermentation management [3], while the resulting wines incur higher duties tax due to their elevated alcohol content. Moreover, the high alcohol content of wine can be a deterrent to consumers who perceive negative health and/or social impacts [4]. There are also wine consumer segments who prefer lighter styles of wine, e.g., wines which typically have lower alcohol levels [2,4]. As such, there is growing interest from the wine industry to better manage wine alcohol levels [4,5].

Harvesting grapes when their sugar content is lower would certainly result in wines of lower alcohol levels. However, this simple approach is not without its drawbacks. Studies have shown that red wine made from grapes harvested earlier—i.e., less mature fruit with lower sugar levels—exhibited less aroma and flavor intensity, especially dark fruit and dried fruit notes, and less pronounced mouthfeel attributes, namely astringency and viscosity, compared to wines made from riper fruit with higher sugar levels [6,7,8]. Instead, these wines were considered to express more red fruit and green aromas and flavors, and higher acidity [6,7,8]. This is likely due to the impact of grape maturity on wine composition. Wines made from less mature fruit usually contain lower levels of grape-derived tannin and yeast-derived mannoproteins (MPs), as well as acetates of higher alcohols derived from yeast sugar and nitrogen metabolism, together with higher levels of organic acids, C6 alcohols and 3-isobutyl-2-methoxypyrazine (in certain grape varieties) [7,9,10,11]. Decreasing wine alcohol content can also impact sensory properties through complex interactions such as the loss of body, decreased solubility of volatile compounds, and increased perception of harshness and astringency [12,13,14,15]. This may negatively affect wine quality, because flavor intensity, body, and mouthfeel are considered to be positive drivers of red wine quality [16]. In warmer climates, such as those experienced in many Australian grape growing regions, development of phenolic and flavor maturity in grapes typically lags behind sugar accumulation [17]. Thus, where early-harvest strategies are employed to manage wine alcohol content, remedial measures are often needed to improve wine quality.

The mouthfeel properties of wine incorporate various tactile responses to certain chemical compounds [18]: Astringency is characterized by the drying and puckering sensations elicited by phenolic compounds and organic acids [19]; while body is defined as the perception of oral viscosity, which depends on alcohol, glycerol, and polysaccharide content [19]. Studies concerning the relationship between wine composition and mouthfeel properties have shown that variation in wine phenolic and polysaccharide composition are highly correlated with perceived astringency and viscosity [6,20,21,22]. Therefore, modifying the macromolecule composition of wine would be expected to present a direct and effective method for modifying wine mouthfeel properties.

Compounds that are native to wine can be isolated from exogenous sources and supplemented into wine at different stages of production. Oenotannin, derived from grape, oak or other plant materials, is one of the more widely used wine supplements [23]. Winemakers use oenotannin to enhance color stability, mask faults, and/or fine-tune wine style [24]. Condensed tannins, extracted from grape seed and skin, are the main phenolic compounds found in red wine. Skin tannins generally have a higher mDP and are rich in epigallocatechin subunits but low in epicatechin-gallate; whereas seed tannins have higher proportions of epicatechin-gallate, no epigallocatechin and lower mDP, despite having marginally higher hydrodynamic volumes (i.e., size, determined by gel permeation chromatography) than skin tannins, at set molecular masses [25]. Together, these attributes can have a significant impact on the perception of astringency [20,26,27]. Oenotannins extracted solely from grape skins or seeds are commercially available, but differences in their sensory impact have not been well studied. Another important class of wine macromolecules used as supplements are mannoproteins (MPs). MPs are proteinaceous polysaccharides derived from yeast cell walls and are the most abundant neutral polysaccharides present in wine [28]. Higher MP concentrations have been associated with decreased ‘green tannin’ characters (harshness, acidity) and enhanced sensations of ‘sweetness’, ‘roundness’, and ‘fullness’ on the palate [22,29,30]. Since wines made from early-harvest grapes are typically low in tannin and MP, direct supplementation of these two classes of compounds might be expected to improve the mouthfeel properties of these wines.

In this study, three winemaking supplements—two oenotannins and a mannoprotein—were added to Shiraz wines post-fermentation. Supplementation of wines made from both unripe (early-harvest) and mature grapes gave a series of wines comprising different ethanol, tannin, and polysaccharide profiles, which enabled interactions attributable to these wine components to be evaluated using sensory analysis techniques. This study specifically aimed to explore the effect of additives on astringency and viscosity, since ethanol, polysaccharides, and tannins, and interactions between these constituents, have previously been shown to affect these sensory characteristics. Whereas previous studies have involved model wine solutions or supplementation during fermentation, the current study investigated the potential for mouthfeel properties to be modified simply by adjusting the concentration of key wine macromolecules in finished wines. The study also sought to determine to what extent the mouthfeel deficiencies perceived in Shiraz wine made from less ripe (early-harvest) grapes could be mitigated through the selective use of commercial supplements.

## 2. Materials and Methods

### 2.1. Grapes and Wine

Shiraz grapes were sourced from a commercial vineyard located in the McLaren Vale region of South Australia (35°17′S, 138°55′E). In 2016, the mean January temperature was 23.4 °C, with the mean maximum temperature exceeding 30 °C on 10 days. Grapes were harvested at two distinct time points: (i) harvest 1 (hereafter H1, on 1 Feb 2016) when the total soluble solids (TSS) content of grapes (400 kg) was 20.8 °Brix; and (ii) harvest 2 (hereafter H2, on 17 Feb 2016) when TSS of grapes (400 kg) was 24.5 °Brix. The weather was mild between the two harvest dates, with daily mean maximum temperatures below 30 °C and only 30 mm of rainfall in total (climate data obtained from www.bom.gov.au). Average berry weights for H1 and H2 were 1.06 and 1.09 g respectively, indicating berry shriveling did not occur between the two harvest dates.

Winemaking, including grape and fermentation analyses, were performed by the WIC Winemaking Service (Urrbrae, SA, Australia). For each harvest date, grapes were divided into two parcels of 200 kg, destemmed and crushed, with samples collected for chemical analysis (Table 1).

After crushing, 5 g of potassium metabisulphite (PMS) and 30 g of EC1118 yeast (Lallemand, SA, Australia) were added to initiate fermentation. Fermentation temperatures were maintained at 15–20 °C, with caps plunged twice daily. After seven days, wines were pressed, fermented to dryness (i.e., <1 g/L residual sugar) and racked. Wines were then inoculated with 0.2 g/L of Lalvin VP41 lactic acid bacteria (Lallemand). On completion of malolactic fermentation, wines were racked off lees and free SO_2_ levels adjusted to 40 mg/L, with samples again collected for chemical analysis (Table 2).

The pH of H1 and H2 wines were adjusted to 3.5 and 3.6 via the addition of 2.5 and 4.0 g/L tartaric acid, respectively. Wines were then cold stabilized at 0 °C for 21 days. Total tannin and polysaccharide levels were measured and found to be similar between wine replicates from the same harvest (data not shown). As such, wine replicates were blended and stored at 0 °C in airtight stainless steel kegs (without ullage), until required.

### 2.2. Addition of Oenotannin and Mannoprotein Supplements

Two oenotannins (one derived from grape skin and one derived from grape seed) and a MP were selected from a range of commercially available supplements, for which compositional data has previously been reported [31]. The selected supplements were chosen based on their compositional similarity to native wine tannin and mannoprotein. The seed tannin was sourced as a dry powder and stored at −20 °C until needed, when it was added to wine without further purification, whereas the skin tannin and MP were liquid products and required modification to obtain powdered forms.

The skin tannin was stored at 4 °C in sealed bottles (as per the manufacturer’s recommendation), prior to modification according to previously published procedures [32]. Non-phenolic material and phenolic compounds of low molecular mass were removed by mixing 1 L of skin tannin with two volumes of Amberlite FPX66 polymeric resin (Dow AgroSciences, NSW, Australia), prewashed with 2 L of 0.5% acetic acid. The mixture was stirred at room temperature for one hour, then filtered through glass wool. The retained resin was washed with 1 L of MilliQ water (containing 0.5% acetic acid) and then 4 L of 50% methanol (containing 0.5% acetic acid), with both fractions being discarded. The polymerized phenolic compounds were then eluted with 2 L of 70% acetone (containing 0.5% acetic acid) and the eluent filtered through a borosilicate glass microfiber filter (0.5 µm, Advantec, John Morris Australia, SA, Australia). The solvent was removed by rotary evaporation at 34 °C, with the pressure gradually being lowered to 30 mbar and then further operated for an hour to ensure complete removal of acetone. The remaining solution was then lyophilized and approximately 8 g of purified tannin was recovered. A sub-sample was dissolved in model wine (12% aqueous ethanol, pH 3.5) and analyzed by HPLC to confirm the absence of residual acetic acid. The remaining purified skin tannin was stored at −20 °C. The MP was purified by dialysis against MilliQ water, using a 7 kDa cut-off membrane (SnakeSkin dialysis tubing, Thermo Scientific, Rockford, USA) against four changes of water, before being lyophilized.

Before supplements were introduced to wines, free SO_2_ levels were measured and were found to be 27.2 and 37.3 mg/L for H1 and H2 wines respectively, indicating spoilage or oxidation had not occurred during storage. The free SO_2_ content of H1 wine was adjusted to 35 mg/L with PMS (10% aqueous solution). Supplements were then added to wines based on gravimetric concentrations (i.e., mg of product per L of wine) to achieve the following treatments:

(1) No additives (control)
(2) 300 mg/L skin oenotannin (skin)
(3) 300 mg/L seed oenotannin (seed)
(4) 400 mg/L mannoprotein (MP400)
(5) 1000 mg/L mannoprotein (MP1000)
(6) 300 mg/L skin oenotannin and 1000 mg/L mannoprotein (skin MP1000)
(7) 300 mg/L seed oenotannin and 400 mg/L mannoprotein (seed MP400)
(8) 300 mg/L seed oenotannin and 1000 mg/L mannoprotein (seed MP1000)

Treatments were applied to H1 and H2 wines (3L per treatment, in duplicate). Wines were allowed to warm to ambient temperature prior to the addition of supplements, with stirring for at least one hour after additions were made to ensure no undissolved powder remained. Wines were then bottled (in 375 mL glass bottles under screw cap) and cellared at 15 °C for three months, prior to sensory and chemical analyses being performed. The remaining wine was sealed in airtight stainless steel kegs without ullage, for use as a base for the preparation of standards used in descriptive analysis.

### 2.3. Chemical Analysis of Wines

Wine ethanol concentrations were determined using an alcolyzer (Anton Paar, Graz, Austria). pH and TA (as g/L tartaric acid) were measured using an autotitrator fitted with an autosampler (Mettler Toledo, SA, Australia).

The total tannin concentrations of H1 and H2 wines were measured as methylcellulose precipitable tannin (MCPT). Based on MCPT results, tannin fractions were isolated from 3 mL of H1 wine and 2 mL of H2 wine using solid phase extraction [33], reconstituted to 10 g/L in methanol and analyzed by phloroglucinolysis to determine subunit composition and the mean degree of polymerization [34,35]. Tannin molecular size was determined by gel permeation chromatography (GPC) using a high-performance liquid chromatograph (HPLC) fitted with a UV–visible detector [32,35]. The percentage of polymeric pigments in the total tannin was estimated based on the ratio between the GPC peak areas recorded at 520 and 280 nm. Wine polysaccharides were isolated from 1 mL of each wine and the composition of monosaccharide residues determined by HPLC using previously published methodology [36].

The composition of each supplement was also determined, using the same methods outlined above. Model wine (12% aqueous ethanol, pH 3.5) solutions of seed and skin oenotannins (1 g/L) were prepared for MCPT determination. Oenotannins were subsequently dissolved in methanol (10 g/L) and subjected to phloroglucinosis and gel permeation chromatography (as above). A model wine solution of 1 g/L MP was prepared for HPLC analysis (as above).

### 2.4. Sensory Analysis of Wines

Wines were subjected to descriptive analysis (DA) with a panel of nine judges (three male, six female) aged between 54 and 70 years old. All judges were initially screened according to ISO standards and had previously completed at least 60 h of red wine-related DA. As such, panelists were familiar with descriptive terms typically associated with red wine. Seven training sessions were held. During the first two sessions, judges familiarized themselves with a range of standards representative of common wine taste and mouthfeel properties, including sweetness, sourness, bitterness, astringency, hotness, and viscosity. In the next two sessions, judges tasted all treatments of H1 and H2 wines, and were asked to discuss and define the mouthfeel sensations perceived. Four terms were defined based on panel consensus: ‘body’ was defined as the perception of viscosity, weight and density; ‘astringency’ was defined as puckering, grippy (drag of the tongue on surfaces of the mouth) and rough sensations; ‘texture’ was defined as sensations of smoothness or coarseness on surfaces of the mouth; and ‘hotness’ which was defined as warm, tingling, and numbing sensations. In the remaining sessions, judges rated a subset of wines using line scales, with results being compared to improve panel agreement/performance. Two descriptive terms, astringency and body, were specifically emphasized by presenting the panel with base wines containing different levels of grape seed extract (to enhance astringency) and xanthan gum (to enhance body) [37]. On completion of training sessions, the ability of the panel to discern variation in astringency was assessed by performing directional paired-comparison tests (in duplicate) using H1 base wine spiked with seed tannin (at 300, 600, and 1000 mg/L). A similar test was performed for ‘body’ (in duplicate) using H1 base wine spiked with xanthan gum (at 350, 500, and 650 mg/L). Panelists then rated ‘astringency’ and ‘body’ (using line scales) for a series of H1 wines spiked with seed tannin (at 300, 600, 1000, and 1500 mg/L) or MP (at 400, 1000, 3000, and 6000 mg/L).

Two formal evaluation sessions were held in a purpose-built sensory laboratory (maintained at 21 °C), during which panelists were presented with 16 wine samples (35 mL) in ISO standard black wine glasses. Wines were presented using a randomized order and complete block design. Sample presentation and data collection were managed using RedJade software (RedJade, Redwood, CA, USA). The evaluation protocol established during training sessions was followed; i.e., panelists took one sip of wine and swirled it in the mouth for 10 s (controlled by a timer) while rating astringency, sweetness, and sourness. The wine was then expectorated and texture was rated. A second sip of wine was taken, swirled for 10 s and bitterness, body, hotness and flavor intensity were rated. Taste and flavor intensity attributes were included in formal evaluations to avoid a ‘dumping’ effect due to the restricted number of attributes, which can otherwise negatively influence sensory ratings [38]. Each attribute was rated on a 10 cm line scale, anchored with ‘low’, ‘medium’, and ‘high’ at 10, 50, and 90%, with the exception of ‘texture’, which was anchored with the descriptors of ‘corn flour’, ‘semolina’, and ‘polenta’, to convey smooth to coarse mouthfeel. A 2 min break was enforced between samples, with a 10 min break enforced after the 5th and 10th samples. Judges were also instructed to consume plain crackers, and to rinse their mouth with pectin solution (1 g/L) and then filtered water during breaks, so as to minimize any sensory carry-over.

Sensory studies were approved by the Human Research Ethics Committee of The University of Adelaide (project No. H-2015-155).

### 2.5. Data Analysis

Chemical data were subjected to one-way analysis of variance (ANOVA) using XLSTAT (version 2015.4.1, VSN International Limited, Herts, UK), while sensory data were analyzed via a mixed-model ANOVA, with samples as fixed factors and panelists as random factors. For both chemical and sensory data, mean comparisons were performed by Fisher’s least significant difference (LSD) multiple-comparison test at 5% level.

## 3. Results and Discussion

Basic chemical analysis of grapes and wine confirmed compositional differences attributable to fruit maturity at H1 and H2—i.e., lower TSS and pH, and higher TA and malic acid levels for fruit at H1 compared with H2 (Table 1)—which corresponded to lower alcohol content and pH, and higher TA, in H1 wine, compared with H2 wine (Table 2). Similar levels of YAN (in fruit), malic acid, residual sugar and VA were observed (in wines), irrespective of harvest date.

### 3.1. Influence of Tannin and Mannoprotein Additions on Wine Composition

MCPT accounted for 33.4 and 74.3% of the dry weight of skin and seed oenotannins respectively. Therefore, theoretically, 300 mg/L supplementation should have resulted in 100 and 223 mg/L increases in wine MCPT for skin and seed tannin additives, respectively. However, irrespective of harvest time, the observed increases ranged from 143 to 237 mg/L for skin tannin addition and from 202 to 388 mg/L for seed tannin addition (Table 3 and Table 4), i.e., increases which were higher than the theoretical values. These increases were consistent with those observed immediately after bottling (Table 3 and Table 4), indicating increases were not related to bottle storage. Rather, differences observed between the theoretical and measured values are likely explained by the different matrices, i.e., Shiraz wine as opposed to the model wine solution in which the oenological tannins were dissolved to measure recovery rates. There are many phenolic and non-phenolic compounds present in red wine, but not in model wine, that might have influenced the absorbance of wine at 280 nm. MCPT values are derived from differences in absorbance at 280 nm before and after precipitation with methylcellulose. Previous research has considered matrix effects on MCPT measurement, but were inconclusive [39]. More detailed analyses were therefore warranted.

The subunit composition of wine tannin was determined by phloroglucinolysis. The percentage yield—i.e., the sum of acid labile subunits—ranged from 15.2 to 23.0%, indicating the subunit values accounted for less than a quarter of the measured MCPT. This result is consistent with previous reports on wine tannin subunit yields [40], because wine tannins are largely resistant to acid hydrolysis and subsequent nucleophilic addition [25]. In H1 wines, treatments involving oenotannin addition gave lower percentage yields than other additives. The percentage yield of purified skin oenotannin determined by phloroglucinolysis was only 7%, likely due to preparation and/or storage conditions. The product was derived from red grape marc post-fermentation which would have been exposed to an oxidative environment before oenotannin was extracted and processed commercially. Furthermore, the oenotannin product was stored as a liquid at 4 °C in a 5 L PE bottle (with 1 L head space) for nine months before being purified for the current study; the pH of the product was 2.34. Both oxygen ingression and low pH environments have been associated with decreased conversion yields in wine tannin, due to changes in tannin structure through oxidation, intra- and inter-molecular bond formation and the incorporation of anthocyanins into tannin polymers [41]. The low conversion yield obtained for the skin oenotannin also explained the lack of any increase in molar proportions of epigallocatechin in supplemented wines. Surprisingly, skin tannin addition did not result in changes in the percentage of polymerized pigment in total tannin either, based on GPC determinations (Table 3 and Table 4). Compared to skin tannin, addition of seed tannin resulted in more apparent modifications to wine tannin composition. In both H1 and H2 wines, treatments involving seed tannin addition resulted in increased epicatechin-gallate subunits and epicatechin terminal subunits, namely those subunits which are typically higher in grape seed tannins [40]. Seed tannin addition also resulted in a slight decrease in mDP, and an increase in tannin molecular mass (determined by GPC), also in agreement with characteristics reported for seed tannins [35].

The MP additive used in the current study yielded 500 mg/L of mannose and 100 mg/L of glucose residues following hydrolysis of 1 g/L of product (in water). Accordingly, a 400 mg/L addition of supplement should theoretically have yielded an increase of 200 mg/L of mannose residues. By extension, 500 mg/L mannose should have been detectable in treatments with 1000 mg/L addition of MP. However, only 55 to 65% of theoretical values were detected in treatments comprising addition of 400 and 1000 mg/L of MP supplement (Table 5), irrespective of any addition of oenotannin or harvest time. These differences might originate from interactions between mannoprotein and other wine components that made them resistant to either precipitation by ethanol or hydrolysis by acid [36].

Despite the somewhat unexpected recoveries of tannin and polysaccharide in experimental treatments, differences observed in wine composition between treatments were still significant, especially in relation to total MCPT and polysaccharide concentrations (Table 3, Table 4 and Table 5). Across the 16 treatments, tannin concentrations ranged from 326 to 1067 mg/L. At these levels, increased tannin concentrations have been shown to be positively associated with perceived astringency [42,43]. In contrast, MP content varied between 72 and 452 mg/L amongst the treatments. A previous study found that MP was negatively correlated with astringency in a red wine, at concentrations between 75.2 and 186.2 mg/L (concentrations typically found in red wines) [20]. Vidal and colleagues found that a blend of MP and arabinogalactan (at 500 mg/L) elicited full and rounded mouthfeel sensations in model wine [29]. It was therefore expected that in the current study, wines would differ in their mouthfeel properties given the large variation in their tannin and MP levels.

### 3.2. Influence of Tannin and Mannoprotein Additions on Wine Sensory Properties

#### 3.2.1. Sensory Profiles of H1 and H2 Wines Following Addition of Wine Supplements

**Figure 1:**
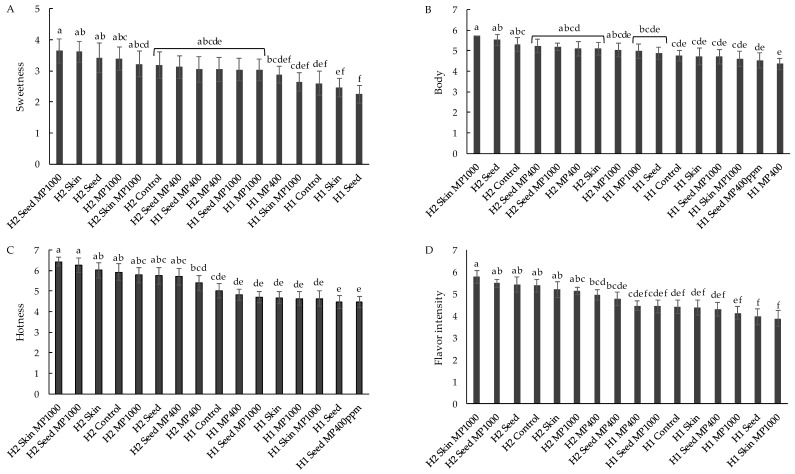
Intensity of selected sensory descriptors, (**A**) sweetness, (**B**) body, (**C**) hotness and (**D**) flavor intensity, for H1 and H2 wines following the addition of tannin and/or mannoprotein (MP) supplements. Letters indicate significant differences (*p* ≤ 0.05, mixed model ANOVA, post hoc Fisher’s LSD).

**Figure 2:**
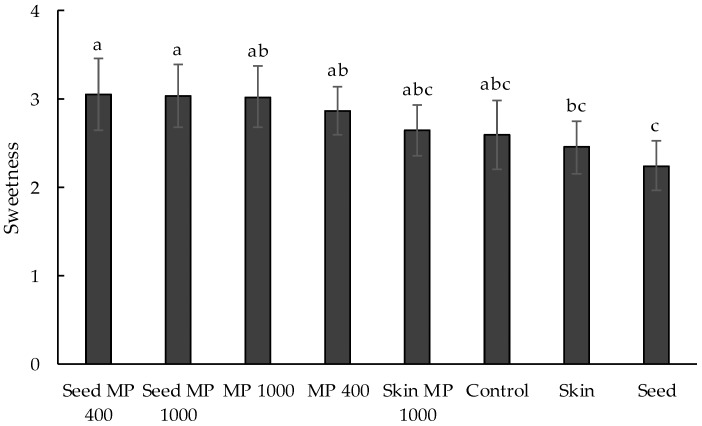
Intensity of sweetness for H1 wines made with the addition of tannin and/or mannoprotein (MP) supplements, either individually or in combination. Letters indicate significant differences (*p* ≤ 0.05, mixed model ANOVA, post hoc Fisher’s LSD).

Of the sensory descriptors evaluated by descriptive analysis, four were found to be significantly different for H1 and H2 wines, being: ‘sweetness’, ‘body’, ‘hotness’, and ‘flavor intensity’ (Figure 1). The intensity of each of these attributes were rated higher in H2 wines than in H1 wines, with the exception of sweetness, which was perceived to be higher in H1 Seed MP400 than in H2 MP400 (Figure 1A). These results are in agreement with previous studies that explored the impact of fruit maturity at harvest on wine sensory properties [6,11]. By comparison, the effect of supplementation amongst sets of wines (i.e., H1 wines or H2 wines) was very small; only the ‘sweetness’ of H1 wines was perceived to be significantly different, with the five treatments involving MP addition rated sweeter than control wines and wines supplemented with skin or seed tannin (Figure 2). MP has been found to increase the perception of sweetness or to suppress bitterness of grape derived tannins [30,44]. However, in the current study, there was no relationship between sweetness and the level of MP added, nor whether MP was used individually or in combination with oenotannin. Oenological tannin addition has been shown to enhance the perception of bitterness [45,46], but this was not observed in the current study. However, addition of oenotannins may still have diminished the perception of sweetness. Thus, it is possible that the perceived differences in sweetness actually reflected increased bitterness due to oenological tannin treatments. No differences in sweetness were found in H2 wines, which might reflect an associated elevation in sweetness intensity (Figure 1A), i.e., any effects of oenotannin and MP were negated by increased fruit ripeness.

#### 3.2.2. Influence of Oenotannin Supplementation on Perceived Astringency in Wine

**Figure 3:**
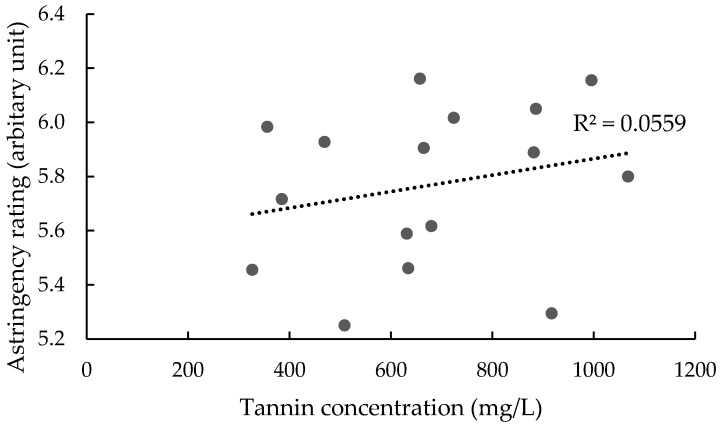
Intensity of astringency as a dependent variable of tannin concentration; dots represent treatments. A linear trend line was fitted to all data points, with the linear coefficient (*R*^2^) shown.

**Figure 4:**
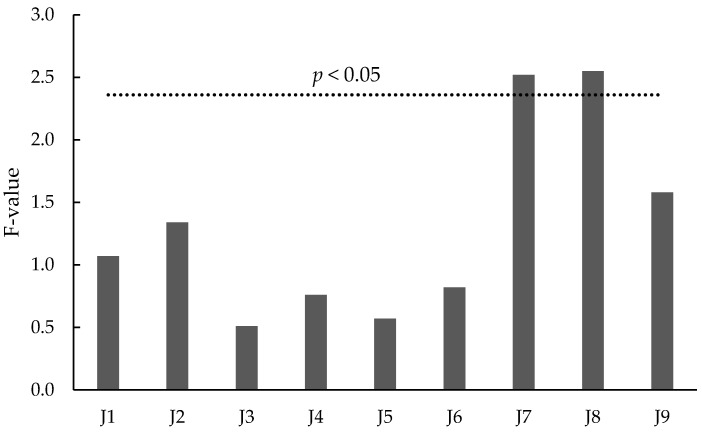
F-values for astringency ratings by individual judge (J1–J9); where bars exceed the dotted line, judges could differentiate astringency levels amongst samples.

**Figure 5:**
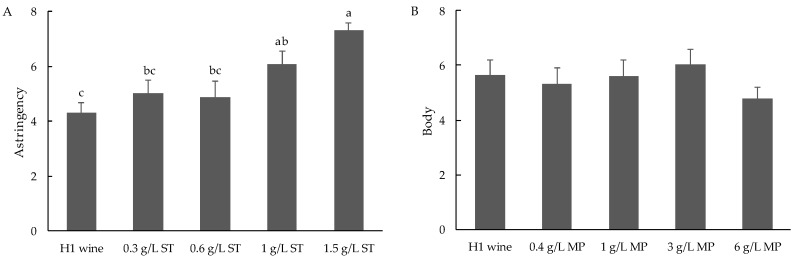
Ratings for ‘astringency’ and ‘body’ of H1 wines spiked with (**A**) 0.3 to 1.5 g/L seed tannin and (**B**) 0.4 to 6 g/L mannoprotein (MP), respectively. Letters indicate significant differences.

Surprisingly, no relationship was found between tannin concentration and astringency ratings (Figure 3). The replicate effect was not significant for astringency ratings (data not shown). However, of the nine judges, only two could differentiate samples based on astringency (Figure 4). Prior to commencement of formal DA evaluations, the assessors’ ability to differentiate different levels of astringency had been tested through directional paired comparison tests, using H1 base wines spiked with seed tannin (at 300, 600, and 1000 mg/L) as reference standards. Only two judges gave correct responses at all three dose rates; five judges gave correct responses at 600 and 1000 mg/L additions, while two additional judges correctly identified only the 1000 mg/L addition. Panel performance was further assessed by rating astringency (on a line scale) in H1 wines spiked with seed tannin from 300 to 1500 mg/L. Wines spiked with 1000 and 1500 mg/L of seed tannin were perceived to be significantly more astringent than wines spiked with lower tannin levels, which were not perceived to be different from the control (Figure 5A).

It therefore appeared that the judges were able to perceive different levels of astringency, but not within the concentration range required for wines presented in the current study. Most previous studies have found that astringency ratings increase with increasing wine tannin concentrations [39,42,43,47,48]. However, some studies reported the perception of astringency had a relatively weak correlation with tannin concentration, and was instead driven by tannin subunit composition, the degree of polymerization, hydrodynamic volume, structural conformation, and the degree of anthocyanin incorporation [26,27,49,50]. These parameters were not considerably different among treatments in the current study. Thus, no definitive conclusion regarding any implications for wine astringency could be drawn from tannin compositional data.

It is also possible that the differences in tannin concentrations amongst treatments (being 200–600 mg/L) were simply too low for the DA panel to detect as perceptible sensory differences. Landon and colleagues [48] reported astringency differences between wines of low and high tannin concentrations (i.e., 250 vs. 1071 mg/L), but at medium levels (i.e., 631 mg/L), astringency was not significantly different to either the low or high levels. Furthermore, although one study did report a linear increase in astringency with increasing red wine tannin concentration [43], and at levels similar to those reported in the current study, the quantification method used was based on protein precipitation (BSA). A recent review found that although the results from BSA and MCPT were highly correlated, the BSA method gave consistently lower values than MCPT [51]. Therefore, should MCPT have been performed on the wines of the aforementioned study, tannin concentrations may have been much higher. In support of this observation, a linear relationship has been observed between wine MCPT concentration and astringency [39], but with a wider and higher concentration range than presented in the current study. In fact, in the 33 studies published prior to 2016, the MCPT values reported for some 281 wines ranged from 60 to 3530 mg/L, with a median concentration of 1340 mg/L [51]. This suggests that the wines from the current study (including those involving the addition of oenotannin) only represented red wines with minimal to low total tannin concentrations. Thus, differences in astringency may have been too subtle for the DA panel to distinguish.

The overall wine matrix is another factor to consider. The largest difference in tannin concentration was observed between the H1 control and the H2 Seed MP1000 wines, which also differed in terms of ethanol content, polysaccharide content, and perceived sweetness; factors which are all expected to affect the perception of astringency [20,44,52]. Notwithstanding, a previous study has demonstrated a significant difference in astringency in Cabernet Sauvignon wines made from fruit harvested at different levels of maturity, with MCPT ranging from 731 to 1088 mg/L [6,10]. Thus, it is entirely possible that the current panel simply did not possess the sensitivity to distinguish subtle levels of astringency.

#### 3.2.3. Influence of Mannoprotein Supplementation on Perceived Body and Astringency in Wine

The addition of MP did not decrease the perception of astringency in wine, despite some treatments containing over 400 mg/L mannose residues, i.e., levels far exceeding what is typically observed in red wine (80 to 200 mg/L) [10,11,20,21]. Mannoprotein has been shown to limit seed tannin aggregation [53] and mediate tannin and protein interactions [54], thereby potentially having the ability to modulate wine astringency. Reductions in astringency have been inferred through reduction of the gelatin index following the addition of MP to polyphenols [55] or through establishing negative correlations between MP concentrations and astringency ratings, using multivariate analysis [20]. The addition of MP, at much lower levels than those used in this study, during vinification has been shown to reduce astringency and/or harsh tannin characters in wine [22,36,56]. However, in these studies, racking and/or filtration processes were employed after the addition of MP. Thus, it is not certain to what extent fining achieved the observed reduction in astringency. Another study in which MP was directly added to model wine containing 250–750 mg/L of tannin did not achieve any reduction in the perception of astringency [44], which was in agreement with findings from this study. Clearly the purported effect of MP on astringency requires further investigation.

The mouthfeel perception, ‘body’, was also explored in this study. Directional paired comparison tests using H1 base wine spiked with xanthan gum (at 350, 500, and 650 mg/L) were used to evaluate each panelist’s ability to perceive body [37]. Five judges gave correct responses at all concentrations, with an additional two judges correct at both 500 and 650 mg/L levels. The panel found differences in body between H1 and H2 wines (Figure 1). However, there was no effect on ‘body’ for treatments involving MP supplementation, in either H1 or H2 wines. Furthermore, when rating ‘body’ for a series of H1 base wines spiked with MP (at 400, 1000, 3000, and 6000 mg/L), no significant differences (or trends) were identified by the DA panel (Figure 5B). The panel could differentiate ‘body’ as a mouthfeel property, but they did not relate it to any sensory impact arising from MP addition. Wine body is usually classified as light, medium, or full, but these terms are very loosely defined in wine sensory evaluation [19]. In a frequently cited red wine mouthfeel wheel, the definition of ‘thin’ was “low in body, viscosity and flavor” [57]. Similarly, in practice, ‘wine body’ was found to be related to viscosity, flavor perception and overall intensity of wine [37,58]. The multi-modal sensory interactions were supported by observations that the perception of ‘body’ decreased in wine made from early-harvest grapes compared to wines made from riper fruit [6], or after a wine was dealcoholized [14]. In the current study, perceived differences in ‘body’ were concurrent with differences in ‘sweetness’, ‘hotness’, and ‘flavor intensity’ amongst H1 and H2 wines (Figure 1). Therefore, it is possible that differences in body were driven by interactions. The addition of MP to H1 wines may well have yielded a sensory impact, but this was not recognized as ‘body’ by the DA panel. Wine neutral polysaccharides, including arabinogalactan-protein and mannoprotein, have been demonstrated to illicit ‘fullness’ sensations in model wine [29] and to increase the viscosity of white wine [59]. However, no direct effect of MP on body in red wine has been reported, and the current study did not find any contribution of MP to red wine body. Further investigations are therefore warranted. However, given that an upper limit of 400 mg/L of MP addition is imposed in Australian wines, based on an agreement between Australia and the European Community on Trade in Wine [60], it is likely that the contribution of MP to red wine body might not be applicable to winemaking settings in this context.

## 4. Conclusions

Two oenotannins (derived from grape seed and skin) and a mannoprotein were added (at different rates and/or in combination) to Shiraz wines made from fruit harvested at two distinct levels of maturity. This gave rise to a series of wines with significantly different tannin, mannoprotein, and ethanol concentrations. A trained panel perceived sensory differences between H1 and H2 wines, but could not perceive any effect of the supplements on wine sensory properties, with the exception of a minor increase in sweetness attributed to mannoprotein addition to H1 wines. Neither a reduction in astringency nor an increase in body were perceived, even when MP was added to wines at a dose rate 2.5 times higher than the level permitted for use in Australia.

## Figures and Tables

**Table 1:** Composition of grapes harvested at H1 (early) and H2 (maturity).

Harvest	TSS (°Brix)	pH	TA ^1^ (g/L)	Malic Acid (g/L)	YAN ^2^ (g/L)
H1	20.8	3.5	5.2	3.5	0.21
H2	24.5	3.7	4.4	2.9	0.25

^1^ TA = titratable acidity (as g/L of tartaric acid); ^2^ YAN = yeast assimilable nitrogen. Values are means of two replicates (*n* = 2).

**Table 2:** Composition of wines made from grapes harvested at H1 (early) and H2 (maturity).

Harvest	Alcohol (% *v*/*v*)	pH	TA ^1^ (g/L)	Malic Acid (g/L)	Residual Sugar (g/L)	VA ^2^ (g/L)
H1	11.5	3.9	4.9	<0.05	0.3	<0.25
H2	14.5	4.2	4.5	0.08	0.1	0.32

^1^ TA = titratable acidity (as g/L of tartaric acid); ^2^ VA = volatile acidity (as g/L of acetic acid). Values are means of two replicates (*n* = 2).

**Table 3:** Phenolic composition of H1 wines following the addition of tannin and/or mannoprotein (MP) supplements.

Measurement	Control	Skin	Seed	MP400	MP1000	Skin MP1000	Seed MP400	Seed MP1000	*p*
Total tannin after bottling (mg/L)	329 ± 10 ^c^	557 ± 9 ^a^	608 ± 36 ^a^	381 ± 20 ^c^	373 ± 4 ^c^	476 ± 7 ^b^	608 ± 3 ^a^	605 ± 2 ^a^	*<0.001*
Total tannin (mg/L)	326 ± 5 ^d^	469 ± 56 ^bc^	665 ± 22 ^a^	356 ± 32 ^d^	385 ± 14 ^cd^	508 ± 38 ^b^	634 ± 36 ^a^	631 ± 13 ^a^	*0.001*
Extension subunits ^1^									
C	3.7 ± 0.1^c^	4.3 ± 0.3 ^bc^	6.5 ± 0.2 ^a^	4.4 ± 0.3 ^bc^	4.3 ± 0.1 ^bc^	4.5 ± 0.2 ^b^	6.8 ± 0.2 ^a^	6.6 ± 6.6 ^a^	*<0.001*
EC	47.6 ± 0.0 ^bcd^	49.8 ± 0.4 ^a^	47.8 ± 0.8 ^bc^	48.4 ± 0.9 ^ab^	47.1 ± 1.5 ^bcd^	48.1 ± 0.3 ^bc^	47.5 ± 0.3 ^bcd^	46.3 ± 0.4 ^d^	*0.013*
EGC	26.3 ± 0.8 ^a^	25.5 ± 0.2 ^a^	20.4 ± 0.1 ^b^	25.2 ±0.2 ^a^	26.6 ± 0.9 ^a^	26.2 ± 0.4 ^a^	20.3 ± 0.4 ^b^	21.5 ± 0.2 ^b^	*<0.001*
EC-G	1.7 ± 0.1 ^bc^	1.6 ± 0.1 ^bc^	3.1 ± 1.8 ^a^	1.8 ± 0.0 ^b^	1.4 ± 0.1 ^c^	1.5 ± 0.1 ^bc^	2.8 ± 0.1 ^a^	2.8 ± 0.1 ^a^	*<0.001*
Terminal subunits ^1^									
C	16.2 ± 0.5	13.9 ± 0.1	14.4 ± 0.4	15.7 ± 0.7	15.5 ± 0.0	14.5 ± 0.2	14.8 ± 0.2	14.8 ± 0.5	*0.089*
EC	4.8 ± 0.2 ^b^	4.7 ± 0.1 ^b^	7.2 ± 0.2 ^a^	4.4 ± 0.4 ^b^	4.7 ± 0.3 ^b^	5.1 ± 0.1 ^b^	7.2 ± 0.3 ^a^	7.6 ± 0.2 ^a^	*<0.001*
EC-G	0.2 ± 0.0 ^cd^	0.2 ± 0.0 ^c^	0.6 ± 0.0 ^a^	0.1 ± 0.0 ^cd^	0.2 ± 0.0 ^cd^	0.1 ± 0.1 ^d^	0.5 ± 0.1 ^ab^	0.4 ± 0.1 ^b^	*<0.001*
% Yield ^2^	22.0 ± 0.4 ^a^	15.2 ± 0.1 ^b^	16.0 ± 0.1 ^b^	21.2 ± 2.2 ^a^	18.6 ± 1.1 ^ab^	16.1 ± 1.5 ^b^	17.1 ± 1.3 ^b^	16.6 ± 0.8 ^b^	*0.021*
mDP	4.84 ± 0.16 ^bcd^	5.32 ± 0.03 ^a^	4.51 ± 0.12 ^cde^	4.96 ± 0.28 ^abc^	4.89 ± 0.09 ^abcd^	5.08 ± 0.08 ^ab^	4.44 ± 0.05 ^de^	4.38 ± 0.14 ^e^	*0.014*
Tannin molecular mass (g/mol) ^3^	1670 ± 49 ^bc^	1745 ± 30 ^bc^	1931 ± 81 ^a^	1644 ± 58 ^c^	1621 ± 32 ^c^	1673 ± 49 ^bc^	1836 ± 55 ^ab^	1815 ± 50 ^ab^	*0.027*
% Colored (520:280) ^4^	14.6 ± 0.1	14.7 ± 0.1	11.7 ± 0.0	12.1 ± 2.2	14.3 ± 0.0	14.2 ± 0.3	11.5 ± 0.2	11.7 ± 0.2	*0.05*

Values reported in the first row were derived from measurements performed on wines immediately after bottling. Values are means of 2 replicates ± standard error. Values followed by different letters within rows are significantly different (*p* ≤ 0.05, one-way ANOVA, post hoc Fisher’s LSD). ^1^ Molar proportion of subunit composition determined by phloroglucinolysis. C = catechin; EC = epicatechin; EGC = epigallocatechin; and EC-G = epicatechin-gallate. ^2^ %Yield determined by dividing the total concentration of individual subunits by the concentration of tannin used in phloroglucinosis reaction. ^3^ Determined by gel permeation chromatography at 50% elution. ^4^ GPC peak area at 520 nm as a percentage of peak area at 280 nm.

**Table 4:** Phenolic composition of H2 wines following the addition of tannin and/or mannoprotein (MP) supplements.

Measurement	Control	Skin	Seed	MP400	MP1000	Skin MP1000	Seed MP400	Seed MP1000	*p*
Total tannin after bottling (mg/L)	686 ± 21 ^d^	824 ± 12 ^bc^	903 ± 12 ^ab^	733 ± 65 ^cd^	761 ± 18 ^cd^	825 ± 6 ^bc^	1006 ± 52 ^a^	976 ± 4 ^a^	*0.001*
Total tannin (mg/L)	679 ± 11 ^d^	916 ± 10 ^c^	995 ± 17 ^b^	724 ± 20 ^d^	657 ± 17^d^	886 ± 10 ^c^	881 ± 14 ^c^	1067 ± 47 ^a^	*<0.001*
Extension subunits ^1^									
C	4.0 ± 0.5	4.4 ± 0.3	5.1 ± 0.4	4.2 ± 0.5	4.1 ± 0.5	4.3 ± 0.1	5.2 ± 0.0	5.4 ± 0.3	*0.165*
EC	48.8 ± 1.7 ^ab^	49.8 ± 0.1 ^a^	50.1 ± 0.0 ^a^	49.0 ± 1.1 ^ab^	46.7 ± 0.4 ^bc^	45.7 ± 0.5 ^c^	46.4 ± 0.8 ^bc^	46.4 ± 0.9 ^bc^	*0.032*
EGC	29.0 ± 2.2 ^ab^	28.2 ± 0.6 ^b^	23.7 ± 1.3 ^c^	28.2 ±1.0 ^b^	30.1 ± 0.6 ^ab^	32.1 ± 0.1 ^a^	26.6 ± 0.0 ^bc^	27.6 ± 1.1 ^b^	*0.018*
EC-G	2.1 ± 0.0 ^cd^	2.1 ± 0.1 ^cd^	2.9 ± 0.1 ^a^	2.2 ± 0.0 ^c^	2.1 ± 0.0 ^cd^	1.9 ± 0.0 ^d^	2.8 a ± 0.1 ^a^	2.4 ± 0.1 ^b^	*<0.001*
Terminal subunits ^1^									
C	11.2 ± 0.0	10.7 ± 0.2	12.0 ± 0.5	11.3 ± 0.3	11.8 ± 0.4	10.8 ± 0.3	11.9 ± 0.2	11.5 ± 0.1	*0.085*
EC	4.8 ± 0.1 ^b^	4.8 ± 0.3 ^b^	6.1 ± 0.3 ^a^	5.0 ± 0.2 ^b^	5.1 ± 0.0 ^b^	5.2 ± 0.2 ^b^	6.9 ± 0.4 ^a^	6.5 ± 0.3 ^a^	*0.002*
EC-G	nd	nd	0.2 ± 0.0 ^a^	nd	nd	nd	0.2 ± 0.0 ^a^	0.2 ± 0.0 ^b^	*<0.001*
% Yield ^2^	19.2 ± 3.9	18.2 ± 1.7	17.4 ± 0.6	21.1 ± 1.2	21.9 ± 1.3	18.1 ± 0.0	23.0 ± 0.9	18.3 ± 1.9	*0.330*
mDP	6.23 ± 0.01 ^ab^	6.46 ± 0.20 ^a^	5.47 ± 0.24 ^cd^	6.12 ± 0.17 ^ab^	5.89 ± 0.13 ^bc^	6.27 ± 0.20 ^ab^	5.28 ± 0.19 ^d^	5.50 ± 0.13 ^cd^	*0.009*
Tannin molecular mass (g/mol) ^3^	1746 ± 3	1807 ± 26	1889 ± 19	1696 ± 54	1735 ± 41	1746 ± 75	1811 ± 12	1837 ± 52	*0.132*
% Colored (520:280) ^4^	12.9 ± 0.1 ^ab^	13.0 ± 0.1 ^a^	11.4 ± 0.1 ^c^	12.7 ± 0.1 ^b^	12.8 ± 0.1 ^ab^	13.0 ± 0.0 ^a^	11.3 ± 0.0 ^c^	11.4 ± 0.0 ^c^	*<0.001*

Values reported in the first row were derived from measurements performed on wines immediately after bottling. Values are means of two replicates ± standard error. Values followed by different letters within rows are significantly different (*p* ≤ 0.05, one-way ANOVA, post hoc Fisher’s LSD); nd = not detected. ^1^ Molar proportion of subunit composition determined by phloroglucinolysis. C = catechin; EC = epicatechin; EGC = epigallocatechin; and EC-G = epicatechin-gallate. ^2^ %Yield determined by dividing the total concentration of individual subunits by the concentration of tannin used in phloroglucinosis reaction. ^3^ Determined by gel permeation chromatography at 50% elution. ^4^ GPC peak area at 520 nm as a percentage of peak area at 280 nm.

**Table 5:** Total polysaccharide concentrations, and monosaccharide residues (mannose and glucose) after hydrolysis, for H1 and H2 wines following the addition of tannin and/or mannoprotein (MP) supplements.

Measurement	Control	Skin	Seed	MP400	MP1000	Skin MP1000	Seed MP400	Seed MP1000	*p*
H1 wines									
Total polysaccharides (mg/L)	401 ± 45 ^cd^	387 ± 3 ^d^	398 ± 4 ^d^	520 ±51 ^b^	801 ± 15 ^a^	755 ± 24 ^a^	495 ± 18 ^bc^	754 ± 29^a^	*<0.001*
Mannose ^1^	72 ± 7 ^c^	68 ± 1 ^c^	69 ± 0 ^c^	186 ± 18 ^b^	409 ± 11 ^a^	378 ± 9 ^a^	173 ± 8 ^b^	397 ± 10 ^a^	*<0.001*
Glucose ^2^	20 ± 2 ^c^	21 ± 0 ^c^	21 ± 0 ^c^	40 ± 4 ^b^	80 ± 0 ^a^	78 ± 5 ^a^	37 ± 1 ^b^	71 ± 4 ^a^	*<0.001*
H2 wines									
Total polysaccharides (mg/L)	403 ± 4 ^bcd^	395 ± 9 ^cd^	383 ± 23 ^d^	518 ± 27 ^b^	720 ± 28 ^a^	782 ± 67 ^a^	504 ± 14 ^bc^	820 ± 64 ^a^	*<0.001*
Mannose ^1^	98 ± 2 ^cd^	92 ± 3 ^d^	91 ± 6 ^d^	214 ± 10 ^b^	404 ± 4 ^a^	432 ± 36 ^a^	210 ± 7 ^bc^	452 ± 42 ^a^	*<0.001*
Glucose	19 ± 1 ^b^	24 ± 3 ^b^	20 ± 1 ^b^	29 ± 2 ^b^	53 ± 4 ^a^	60 ± 6 ^a^	27 ± 3 ^b^	62 ± 2 ^a^	*<0.001*

Values are means of two replicates ± standard error. Values followed by different letters within rows are significantly different (*p* ≤ 0.05, one way ANOVA, post hoc Fisher’s LSD). ^1^ Concentrations of mannose and glucose residues contained in polysaccharide were determined after hydrolysis. ^2^ Total polysaccharide content was determined as the sum of all monosaccharide residues after hydrolysis, including arabinose, fucose, galactose, galacturonic acid, glucose, mannose, rhamnose, and xylose.
